# Systematic Review of Vitamin D and Hypertensive Disorders of Pregnancy

**DOI:** 10.3390/nu10030294

**Published:** 2018-03-01

**Authors:** Karen M. O’Callaghan, Mairead Kiely

**Affiliations:** 1 Cork Centre for Vitamin D and Nutrition Research, School of Food and Nutritional Sciences, University College Cork, Cork T12 Y337, Ireland; karen.ocallaghan@ucc.ie; 2 Irish Centre for Fetal and Neonatal Translational Research (INFANT), University College Cork, Cork T12 DFK4, Ireland

**Keywords:** 25-hydroxyvitamin D, gestational hypertension, preeclampsia, vitamin D

## Abstract

This narrative systematic review evaluates growing evidence of an association between low maternal vitamin D status and increased risk of hypertensive disorders. The inclusion of interventional, observational, and dietary studies on vitamin D and all hypertensive disorders of pregnancy is a novel aspect of this review, providing a unique contribution to an intensively-researched area that still lacks a definitive conclusion. To date, trial evidence supports a protective effect of combined vitamin D and calcium supplementation against preeclampsia. Conflicting data for an association of vitamin D with gestational hypertensive disorders in observational studies arises from a number of sources, including large heterogeneity between study designs, lack of adherence to standardized perinatal outcome definitions, variable quality of analytical data for 25-hydroxyvitamin D (25(OH)D), and inconsistent data reporting of vitamin D status. While evidence does appear to lean towards an increased risk of gestational hypertensive disorders at 25(OH)D concentrations <50 nmol/L, caution should be exercised with dosing in trials, given the lack of data on long-term safety. The possibility that a fairly narrow target range for circulating 25(OH)D for achievement of clinically-relevant improvements requires further exploration. As hypertension alone, and not preeclampsia specifically, limits intrauterine growth, evaluation of the relationship between vitamin D status and all terms of hypertension in pregnancy is a clinically relevant area for research and should be prioritised in future randomised trials.

## 1. Introduction

Hypertensive disorders of pregnancy are a major cause of maternal and foetal severe acute morbidity, long-term disability, and mortality. On a global basis, an estimated 10% of pregnant women suffer from hypertensive disorders, representing a serious threat to public health [1]. Hypertension in pregnancy can be classified by the terms chronic hypertension, gestational hypertension, preeclampsia (PE), or chronic hypertension with superimposed PE [2]. Chronic hypertension refers to a systolic blood pressure (SBP) ≥ 140 mmHg and/or a diastolic blood pressure (DBP) ≥90 mmHg, on at least two occasions, 4 h apart, which predates pregnancy or occurs before the 20th week of gestation. If this hypertension develops after 20 gestational weeks, it is referred to as gestational hypertension or pregnancy-induced hypertension (PIH). The presence of proteinuria has been a long-standing criterion used to distinguish PE from PIH. However, proteinuria has been questioned as a prerequisite in recent years [3], and new diagnostic criteria allow for the identification of PE based on new onset hypertension in the absence of proteinuria, but in combination with haematological abnormalities, renal and liver impairment, neurological symptoms, and uteroplacental dysregulation [2,3]. Where PE occurs in women with longstanding hypertension, this is termed chronic hypertension with superimposed PE [2].

Affecting an estimated 3–5% of pregnancies worldwide [4], PE is a heterogeneous disorder that is alleviated only after delivery of the placenta [5]. Globally, PE accounts for more than 70,000 maternal and over 500,000 infantile deaths per annum [6], and treatment of PE in nulliparous women results in a yearly economic burden of €31 million in the developed world alone [7]. The etiology of PE is not fully discerned and its rapid progression and multisystem involvement challenges the understanding of both the pathogenesis of PE and the development of preventative measures [8]. Initiation of PE is believed to stem from the immune rejection of cytotrophoblasts during placentation, causing impaired remodelling of the spiral arteries of the decidua and myometrium [9]. In recent years, the discovery of vitamin D-specific receptors and metabolites in the placenta and decidua [10] has highlighted a potential role for vitamin D in perinatal health, outside of its established role in skeletal mineralisation [11]. 

Maternal vitamin D metabolism is altered during pregnancy, leading to increased circulating levels of both the vitamin D binding protein (VDBP) [12] and the active metabolite, 1,25-dihydroxyvitamin D (1,25(OH)_2_D) [13]. At term, expectant mothers have almost twice the concentration of 1,25(OH)_2_D compared to non-pregnant women [13], of which at least 50% is thought to be contributed by the placenta and/or decidual tissue [10,14,15]. The precise function of this increase in 1,25(OH)_2_D has not been fully established, but current concepts propose that the surge in 1,25(OH)_2_D is a physiological response induced to permit immune tolerance through vitamin D pathways at the maternal-foetal interface, thereby supporting proper placentation [16].

Widespread vitamin D deficiency has been reported among gravidae worldwide [17], and the potential of vitamin D to prevent pregnancy-related complications is an area of current focus, however, conclusive evidence from randomised trials to support a role for vitamin D in perinatal health is still pending [18]. Women with PE have been shown to experience alterations in calcium and vitamin D metabolism [19]. In comparison to the normal placenta, mRNA expression for the vitamin D-metabolising enzymes CYP2R1, CYP27B1, CYP24A1, and the vitamin D receptor (VDR) have been increased and decreased in placentas of women with PE [20,21,22], providing direct evidence for disrupted vitamin D metabolic homeostasis in the preeclamptic placenta [22]. The underlying mechanism for this disruption and its association with PE development is not fully understood. It is hypothesised that low-circulating 1,25(OH)_2_D leads to an imbalance in immune function, resulting in a shift towards a pro-inflammatory environment [16] and disrupted implantation [19]. Increased tumour necrosis factor (TNF)-α stimulates catabolism of 1,25(OH)_2_D, contributing to the lower circulating calcium levels that are observed in PE-diagnosed pregnancies [23,24]. To state that malplacentation results principally from the pro-inflammatory environment induced by Th1 cytokine activity would be considered a narrow interpretation, however, and it is likely that vitamin D may contribute to multiple innate and adaptive immune responses in placental and decidual tissue [16]. Despite the advances made in vitro, association studies of vitamin D and PIH can be difficult to interpret at a clinical level, owing to the uncertainty of the pathogenesis of PE, alongside the multiple roles of vitamin D in immune function [25].

## 2. Objectives

The purpose of this narrative systematic review was to investigate the association of vitamin D and risk of hypertensive disorders in pregnancy. To date, most systematic reviews have focused on one particular study design only (observational or intervention) [26,27,28], have specified vitamin D status in the inclusion criteria but excluded studies reporting vitamin D intakes [26,27,29], or have focused on PE but not on other forms of pregnancy-associated hypertension [26,27,29,30]. This review aimed to critically evaluate the findings from both observational and interventional studies, in which either vitamin D status or dietary intakes are assessed and includes all outcomes which fall under the umbrella term of gestational hypertensive disorders. Owing to the mixed nature of the study design (observational or interventional), the marker of exposure (vitamin D intake or status) and the study outcome (blood pressure, PIH or PE), we did not conduct a meta-analysis as part of this review. 

### 2.1. PICO

Population: Apparently healthy women having an uncomplicated pregnancy, not diagnosed with PIH prior to commencement of the study (with the exception of case-control designs) and for whom vitamin D intake or status data is available.

Intervention and comparison: Vitamin D (ergocalciferol or cholecalciferol) versus placebo or dose response. Dietary vitamin D intake and/or 25-hydroxyvitamin D (25(OH)D) status.

Outcome: Recorded blood pressure, PIH or PE (as defined by investigator) before onset or at time of delivery.

### 2.2. Search Methods

The Medline (Pubmed) and EMBASE electronic databases were searched from inception to 11 November 2016. A structured search strategy was devised using key terms selected from the Medical Subject Headings (MeSH) database and related text words for “vitamin D”, “vitamin D deficiency”, “pregnancy”, “preeclampsia”, “hypertension”, and “blood pressure”. Where applicable, bibliographies of selected papers were hand-searched for additional references and assessed for inclusion.

### 2.3. Inclusion Criteria

The following inclusion criteria were applied: full text articles published in the English language; apparently healthy pregnancies before diagnosis of PIH; recorded blood pressure outcome; and studies where the vitamin D intake or status of the mother during pregnancy was available. Studies measuring 25(OH)D specifically, and not 1,25(OH)_2_D, were included owing to the short half-life of 1,25(OH)_2_D, making this metabolite unsuitable for accurately assessing vitamin D status.

### 2.4. Exclusion Criteria

Articles were excluded from the final review if they fell under any of the following categories: postpartum maternal outcomes only; methodological validation papers or papers relating to experimental techniques; genetic studies; animal or in vitro models excluding clinical data; studies reporting biological mechanisms of vitamin D metabolism only; studies that focus solely on populations with gestational diabetes mellitus and studies involving participants diagnosed with any pre-existing metabolic disorder known to interfere with vitamin D metabolism, including chronic kidney disease and liver or gastrointestinal disorders.

### 2.5. Data Collection

Titles and abstracts generated by the search strategy were screened independently by a single reviewer (K.M.O.C.) and relevant articles were identified for further investigation. Studies were included if they reported original data only. Letters, comments, and editorials were excluded, in addition to duplicate articles and narrative review articles. The most recent comprehensive systematic reviews were included, provided a meta-analysis was also reported. The full texts of relevant abstracts were assessed and articles included in the final search were agreed following discussions with a second reviewer (M.K.). To ensure uniformity, for studies reporting 25(OH)D concentrations in ng/mL, values were converted to nmol/L by multiplying by a conversion factor of 2.5, and vitamin D intakes expressed in µg/day were converted to IU/day, for which 1 µg is equivalent to 40 IU. For observational studies reporting both vitamin D status and dietary intake, data relating to 25(OH)D was prioritised in discussion. For intervention trials, the supplemental dose was discussed in preference to achieved 25(OH)D concentrations, to allow for comparison between the administered dose range, intervention duration and incidence of PIH.

For clarity, in the absence of a universal consensus on deficiency and sufficiency thresholds for serum 25(OH)D concentrations, throughout the text we used the Institute of Medicine-assigned cut-offs of <30 nmol/L to define vitamin D deficiency, with low vitamin D status referring to values <50 nmol/L [11], and have specified alternatives as appropriate.

## 3. Results

The initial search identified 190 papers from 1957 to 2016. Once duplicates were removed, 56 texts remained after titles and abstracts were scanned for relevance. Ten papers were identified through hand-searching bibliographies of relevant texts. Results were filtered according to the search criteria, producing a final selection of 49 papers. Three recent systematic reviews and meta-analyses were included, for which we excluded the individual studies incorporated in these reviews, unless they reported an outcome of interest (e.g., vitamin D status or intake/PE or hypertension) that did not feature in the meta-analysis. In this way, we achieved maximum coverage of potentially neglected outcomes. In addition to the three systematic reviews, 34 studies were included in the final review, consisting of three randomised controlled trials (RCT) and 31 observational studies.

**Figure 1:**
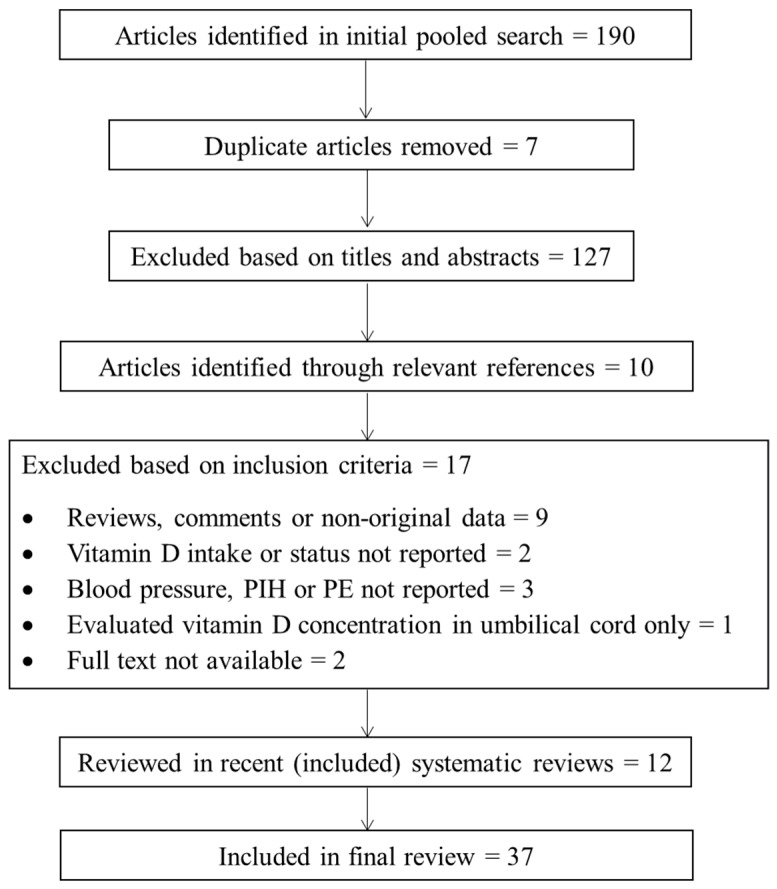
Flowchart of the search strategy and selection process.

A full description of the search strategy and selection process is provided in Figure 1.

### 3.1. Intervention Studies

#### 3.1.1. Preeclampsia

In their updated Cochrane systematic review of RCTs, De-Regil et al. [28] reported the effects of vitamin D interventions on several maternal and infant health outcomes, for which two studies focused on PE. Including a small sample of 219 women, the combined analysis from both trials (one providing 400 IU/day, the other providing up to 4 doses of 120,000 IU across gestation) trended towards reduction of PE with supplementation (8.9% vs. 15.5%; average risk ratio 0.52; 95% confidence interval (CI): 0.25, 1.05) [28]. Using the GRADE (Grading of Recommendations, Assessment, Development, and Evaluation) classification [31], this evidence was deemed of low quality, owing to reporting bias and/or selection, detection, performance, and attrition bias of the two studies. An earlier meta-analysis by Hyppönen and colleagues [30] involved four randomised trials of vitamin D supplementation during pregnancy, of which three were placebo-controlled, unblinded studies (dose range 450–1000 IU/day) and a fourth included a group receiving 400 IU/day as a comparator to the two treatment groups (2000 and 4000 IU/day). This review differed from De-Regil [28] in that a placebo/zero intervention group was not a prerequisite, therefore allowing inclusion of vitamin D dose-comparison studies. A similar conclusion was reached of a reduced risk of PE (pooled odds ratio 0.66; 95% CI: 0.52, 0.83, *p* = 0.001), among supplemented groups. Both meta-analyses highlight insufficient evidence to justify setting a recommendation for supplemental vitamin D during pregnancy based on the avoidance of PE. 

With regard to combined vitamin D and calcium supplementation, the De-Regil et al. [28] meta-analysis of three RCTs (*n* = 1114 women) showed a reduced risk of PE (5% vs. 9%; average risk ratio 0.51; 95% CI: 0.32, 0.80) in supplemented groups. These studies had lower bias than the two small vitamin D only RCTs and were rated as moderate quality evidence. Collectively, the trial evidence to date supports the view that co-supplementation of vitamin D with calcium may reduce the risk of PE more than supplemental vitamin D alone, although this comparison is not evenly matched in terms of sample size or study quality.

#### 3.1.2. Gestational Hypertension

While most published trials focus on development of PE, few have assessed the impact of supplemental vitamin D on the risk of hypertension in pregnancy. We identified three recent trials of vitamin D and gestational blood pressure. Beginning at week 25 of gestation, Asemi [32] noted that a rise in both SBP (−0.2 ± 1.4 vs. 5.5 ± 1.6 mmHg, *p* = 0.01) and DBP (−0.4 ± 1.1 vs. 3.1 ± 1.1 mmHg, *p* = 0.01) was prevented following 9 weeks of vitamin D supplementation (400 IU/day) compared with placebo. This contrasts with findings of an open-label RCT by Hossain [33] in Pakistani women, where supplemental vitamin D did not influence the risk of gestational hypertension relative to routine antenatal care, at a dose corresponding to the current tolerable upper intake level (4000 IU/day) [11]. When the combination of vitamin D plus calcium supplementation was compared to placebo, SBP was unaffected, but a significant decrease was observed for DBP (−1.9 ± 8.3 vs. 3.1 ± 5.2 mmHg, *p* = 0.02) [34], although in a small sample size (*n* = 46) and with a low supplemental dose (200 IU/day). Careful consideration must be given to the sample populations of these 3 intervention studies. In populations of poor socioeconomic status, with a high prevalence of low vitamin D status and where both calcium and vitamin D intakes are likely to be low, correction of vitamin D and calcium deficiency may reduce the incidence of adverse perinatal outcomes. However, the results may not be generally applicable, but are important for vulnerable populations who would benefit from nutritional intervention.

### 3.2. Observational Studies

#### 3.2.1. Vitamin D Status and Preeclampsia

In 2013, Aghajafari et al. [26] published a systematic review and meta-analysis of observational studies, including nine studies that focused on the association of maternal 25(OH)D status and risk of PE. The combined analysis, using the most adjusted model, found PE to be significantly associated with 25(OH)D concentrations <50 nmol/L (pooled odds ratio 1.79; 95% CI: 1.25, 2.58). Heterogeneity between studies was a limiting factor in the meta-analysis, therefore urging caution in the interpretation of the findings. The authors argued that, given the mechanistic underpinning and biological plausibility of the associations between vitamin D and metabolic abnormalities including hypertension, plus the relative consistency in diverse populations where low 25(OH)D levels often precede the adverse outcome (thus reducing the likelihood of reverse causation), their findings may show a causal relationship if examined in appropriately-designed trials [26].

Following this meta-analysis, prospective observational studies continue to examine the role of vitamin D in PE progression. Results from the large, multi-ethnic, seasonally-balanced GraviD study suggest an increase in 25(OH)D concentrations of at least 30 nmol/L from the first to the final trimester is associated with a lower odds of developing PE, irrespective of vitamin D status in early pregnancy. The authors reasoned that 25(OH)D status at the beginning of gestation may not contribute to placental development but that an increment in concentrations may help protect against the initiation of PE as pregnancy progresses towards the latter stages [35]. A similar theory was reported by Wei [36], whose longitudinal assessment of Canadian gravidae found low vitamin D status in the late, but not early, second trimester was associated with a greater risk of PE. The 30 nmol/L increase in 25(OH)D status associated with reduced odds of PE in GraviD corresponds to the observed seasonal variation in vitamin D status in Sweden as well as a lower incidence of PE among women giving birth during summer-autumn compared with winter-early spring [35]. Based on clinical or biochemical assessment, the Evaluating Maternal Markers of Acquired risk of Preeclampsia (EMMA) study identified women (*n* = 221) at high risk of PE in the first half of pregnancy who continued receiving routine antenatal care. Despite the high prevalence (53%) of low vitamin D status, the subsequent risk of PE was not correlated with maternal 25(OH)D [37]. Without measurement of 25(OH)D status at a time closer to delivery, it is not possible to assess whether an increase in vitamin D status from early to late pregnancy would overcome the acknowledged clinical and/or biochemical abnormalities in early pregnancy, therefore, potentially contributing to the hypothesis described by Bärebring [35] and Wei [36]. The EMMA study is also hampered by the heterogeneity of the study sample, in which 10% of women were diagnosed with chronic hypertension at enrolment, introducing significant bias [37]. In a smaller (*n* = 75), less affluent cohort, maternal vitamin D status at delivery was identified as an independent predictor of PE diagnosis among women having a singleton pregnancy and attending a tertiary care facility in Pakistan [38]. However, this sample was malnourished, with 45% having a 25(OH)D concentration <25 nmol/L.

Among a large sample of well-characterised, low-risk nulliparous women, with a 17% prevalence of 25(OH)D <30 nmol/L, we recently reported a 36% reduction in the composite outcome of PE and small-for-gestational-age birth when 25(OH)D concentrations exceeded 75 nmol/L at 15 weeks’ gestation [39]. Similarly, the combination of two prospective cohorts in Canada found 25(OH)D concentrations <30 nmol/L in early pregnancy led to a greater risk of developing PE when compared to concentrations >50 nmol/L [40]. Scholl and colleagues [41] demonstrated a 2.86-fold increased risk of PE in women with elevated parathyroid hormone levels when early pregnancy 25(OH)D was <50 nmol/L. This observation led to the development of the “calcium-metabolic stress” concept, whereby a dysregulation of calcium metabolism, resulting from inadequate dietary calcium and/or low 25(OH)D status, causes secondary hyperparathyroidism in pregnancy which, in turn, increases the risk of PE and hypertensive disorders [41].

In terms of analysing interactions between vitamin D and acknowledged risk factors for PE and pregnancy-induced hypertension, maternal placental growth factor (PlGF) levels were low among women presenting with low vitamin D status in the early and late second trimester [42]. Though both variables were inversely related to PE risk, 25(OH)D and PlGF were not found to share a mutual causal pathway in the development of PE. Therefore, whether the observed association between 25(OH)D and PE risk is linked to impaired angiogenesis [42] is questionable. In a prospective study, biochemical and/or biophysical markers of PE development did not differ among first trimester gravidae with or without low vitamin D status [43]. Nonetheless, retrospective analysis has shown an increased risk of late-onset, but not early-onset, PE among women with low vitamin D status at clinical presentation despite no difference in first trimester 25(OH)D concentrations between PE complicated and non-PE complicated pregnancies [44]. The decrease in 25(OH)D status in late pregnancy among the PE group contrasts to the increase observed for the undiagnosed group, again emphasising the possibility that vitamin D is implicated in PE development in late gestation, independent of differences in the multiple mechanistic pathways of PE pathogenesis in early pregnancy [44].

Apart from longitudinal studies, the relationship between reduced 25(OH)D status and placental biomarkers of PE has been described by Woodham et al. [45] in their nested case-control study of 41 women diagnosed with PE, matched by race/ethnicity to 123 normotensive gravidae at term. In the adjusted regression model, both 25(OH)D concentration and the soluble fms-like tyrosine kinase-1 (sFLT)/PlGF ratio at the beginning of the second trimester were identified as significant predictors of severe PE, for which women experienced pulmonary oedema, seizures, oliguria, or symptoms of hepatic or cerebral dysfunction, in addition to elevated blood pressure and proteinuria. For each 10 nmol/L increase in serum 25(OH)D, a 38% reduction in the odds of developing severe PE was predicted. Similar to that observed in the cohort studies [42,43,44], no interaction was found between 25(OH)D status and developmental biomarkers of PE, therefore extending the theory that vitamin D deficiency and angiogenic factors contribute to PE development through independent mechanistic pathways. Nonetheless, Woodham [45] suggested that combining mid-gestational serum 25(OH)D concentrations with the angiogenic activity factors sFLT and PlGF would yield a better prediction of severe PE than either measure alone. In addition to placental biomarkers, the potential association between vitamin D and markers of oxidative stress [46] and inflammation [47] has also been explored in PE-diagnosed pregnancies at the nested case-control level, both reporting no interaction with 25(OH)D and subsequent PE risk.

Other nested case-control studies provide weak evidence for an association between early pregnancy vitamin D status and PE development, often concluding that low serum 25(OH)D alone does not contribute to PE risk [48,49], even among large, well-characterised sample populations [50]. Subgroup analysis by season of blood collection has shown support for the correlation between low summer time vitamin D status and PE [48]. However, deficiency during the summer months likely indicates lower mean year-round 25(OH)D concentrations and it is plausible that the seasonal vitamin D-PE relationship reflects an association of PE with vitamin D among those who consistently present with low 25(OH)D status. Similarly, in the study by Lechtermann et al. [51], serum 25(OH)D levels stratified by season at delivery were increased only among healthy women giving birth during the summer months, but little response to seasonal variation in vitamin D status was observed for those diagnosed with PE. Moreover, the lack of a seasonal elevation in vitamin D status among women diagnosed with PE may inadvertently reflect lower outdoor physical activity levels, whereby risk of developing a hypertensive disorder is highest among women who are least physically active. In terms of vitamin D metabolism, the negative correlation between CYP27B1 and CYP24A1 that occurs in healthy pregnancies was not evident in the preeclamptic placenta in Lechtermann’s study [51], leading the authors to believe that the dysregulation of CYP24A1 in PE may contribute to the lack of seasonal variation in 25(OH)D status among PE-complicated pregnancies. The small number of patients included in the gene expression analysis (13 cases of PE and 14 controls) questions the reliability of these findings and repetition of this analysis in a larger cohort is required. Though once again limited by sample size (*n* = 48), the work by Anderson and colleagues [52] contrasts to that by Lechtermann [51], in which altered placental gene expression of the VDR and CYP27B1 was identified among women diagnosed with PE compared to controls, for which no difference in mean first trimester 25(OH)D concentrations were observed.

The proposed interplay between angiogenic factors, 25(OH)D concentrations and PE development has also been explored in case-control studies. Despite an almost 50% decrease in median 25(OH)D concentrations among patients with early onset severe PE (EOSPE) compared with gestational age-matched controls, Robinson et al. [53] stressed that 25(OH)D status could not be identified as a stand-alone diagnostic marker, yet deficiency may contribute to the placental modifications that occur during the early stages of PE development. Regarding vitamin D status alone, a lower median 25(OH)D concentration was found among preeclamptic mother-newborn dyads in Brazil [54], and mean serum 25(OH)D concentrations were significantly reduced among PE-diagnosed Iranian women [55,56] and their neonates [56] compared to controls; however, this is likely confounded by the low socioeconomic status and higher risk of PE in deprived communities.

To our knowledge, only one study examined the association between maternal 25(OH)D status and risk of eclampsia specifically [57]. Among a case-control analysis involving 33 diagnoses of PE and 79 diagnoses of eclampsia compared to 76 controls, and following adjustment for age, body mass index (BMI), and pregnancy duration, women with a third trimester plasma 25(OH)D concentration <75 nmol/L exhibited a three- and five-fold increased risk of developing PE and eclampsia, respectively. However, a cut-off of 75 nmol/L is a very conservative level to define vitamin D deficiency; only 12% of women exceeded this threshold. Overall, there was evidence of poor nutritional status, indicated by a low mean BMI (17.7 ± 2.6 kg/m^2^) and a low frequency of supplement use (23%) in the sample population. Nonetheless, a comparison of women in the lowest quartile of 25(OH)D concentrations (<30.1 nmol/L) to those in the highest quartile (>90.2 nmol/L) found the risk of eclampsia was 17 times greater among women with vitamin D deficiency [57]. 

The classification of PE by category, as distinguished by mild (SBP ≥ 140 mmHg and/or DBP ≥ 90 mmHg with proteinuria) or severe (SBP ≥ 160 mmHg and/or DBP ≥ 110 mmHg with elevated proteinuria, plus convulsions or end-organ damage and dysfunction) and its association with vitamin D status, as described by Woodham [45] and Ullah [57], was later explored in two additional case-control designs [58,59]. The analysis by Singla [59], of 74 preeclamptic nulliparous women and 100 controls in India, found maternal 25(OH)D concentrations were negatively associated with blood pressure and risk of PE; however, the severity of PE was not related to vitamin D status. The high prevalence (>80%) of low vitamin D status in this study indicates overall nutritional inadequacy, which was poorly characterised, with no data on maternal anthropometry or prenatal supplement use. It is plausible that the gravidae examined by both Ullah [57] and Singla [59] are at an increased risk of adverse perinatal outcomes irrespective of vitamin D status and, therefore, we caution the extrapolation of these findings to the wider obstetric population. Lastly, Bodnar [58] explored the relationship of vitamin D status and PE risk using data from the multi-ethnic Collaborative Perinatal Project, 1959–1966. Here, low vitamin D status was shown to be a risk factor for severe PE only, but not for milder forms of the disease. PE was most often diagnosed among younger, less educated, black women of poor socioeconomic status. Hence, it is argued that the predictors of PE may correspond to the same predictors of low 25(OH)D status, leading to a biased sample population. Furthermore, the reliability of these results are questionable, as serum 25(OH)D was measured over 40 years after completion of the study [58].

#### 3.2.2. Vitamin D Intake and Preeclampsia

We found only one large prospective cohort that described vitamin D intake in relation to PE. Analysis of vitamin D intakes from over 23,000 nulliparous women participating in the Norwegian Mother and Child Cohort Study [60] found a lower total vitamin D intake was associated with an increased risk of developing PE. Compared to non-supplement users, women taking a vitamin D supplement (400–600 IU/day) had a 27% reduced risk of PE, potentially supporting the theory of a role for vitamin D supplementation in PE prevention, as was discussed earlier [28,30].

#### 3.2.3. Gestational Hypertension

Focusing on gestational hypertension alone, higher plasma 25(OH)D concentrations were associated with a greater risk of hypertension among 1591 women (16.4–36.9 weeks’ gestation) participating in Project Viva [61], corresponding to an odds ratio of 1.32 for each 25 nmol/L increment in 25(OH)D concentration. Though contrary to the current hypothesis, such findings support the previously-reported association of higher vitamin D intakes with increased risk of gestational hypertension from this cohort [62]. Nonetheless, a 25 nmol/L increment is substantial in view of the reported distribution of 25(OH)D in the Project Viva cohort and among pregnant women elsewhere [17,39]. In a much smaller sample (*n* = 75), the prospective cohort of mother-infant dyads by Hossain and colleagues [38], that acknowledged vitamin D status as an independent predictor of PE, also identified maternal 25(OH)D concentration at delivery as an inverse independent predictor of mean arterial pressure, for which, again, the findings are confined to a specific, vulnerable population. Similar caution should be applied to the large (*n* = 1000) cross-sectional study by Al-Shaikh [63], in which there was also a high prevalence of low vitamin D status (86%), and where hypertensive disorders were not seen among women presenting with a 25(OH)D concentration ≥75 nmol/L at delivery. Furthermore, the frequency of PIH did not differ significantly between those with or without low vitamin D status and the fact that over half (57%) the study population were classified as obese upon admission to antenatal care, coupled with an extremely low incidence of PE (<1%), raises concerns as to whether additional cases of PE may have been misdiagnosed or undetected. Irrespective of sample size (*n* ranged from 48 to 263) and diversity of the populations studied, case-control studies stratified by maternal vitamin D status [52], total vitamin D intake [52,64,65], or supplement use [55,64], do not report effects on blood pressure or PIH development.

A summary of observational studies that assessed the risk of gestational hypertensive disorders with serum 25(OH)D status or maternal vitamin D intake is provided in Table 1 and Table 2, respectively. For ease of understanding, an overview of recent systematic reviews that have explored this topic is provided in Table 3.

## 4. Discussion

At present, there is insufficient evidence to justify setting dietary vitamin D recommendations based on the avoidance of gestational hypertensive disorders alone. However, this review supports the findings from two recent meta-analyses of observational studies that have shown a significant association between vitamin D deficiency and PE risk [26,27]. Notwithstanding the controversy regarding appropriate 25(OH)D cut-off points for vitamin D deficiency, both meta-analyses observed a significant correlation when deficiency was defined as 25(OH)D concentrations <50 nmol/L [26,27]. Of note is that, in the subgroup analysis reported by Tabesh [27], the inverse relationship was significant for studies conducted in the United States only, suggesting the vitamin D-PE interaction may be region-specific and reiterates the need for consideration of variation in latitude and ethnicity in vitamin D association studies. 

We identified several challenges in our interpretations. In terms of study design, vitamin D status is often only evaluated at a single time point throughout gestation, frequently in the late second or third trimester, when it may be too late to intervene. It is likely that 25(OH)D measurements throughout each trimester would give a clearer understanding of any potential cause and effect relationship [48] and the most effective strategy for intervention. Furthermore, if 25(OH)D is measured following PE diagnosis, reverse causality cannot be discounted, and the results, therefore, offer limited clinical utility. In addition to analytical differences, the lack of consensus regarding a 25(OH)D cut-off to define vitamin D sufficiency hampers international comparisons of deficiency prevalence and subsequent risk of adverse health outcomes, including PE and gestational hypertension. 25(OH)D data must be reported across all studies using a range of thresholds to facilitate cross-comparison between populations. Based on a 25(OH)D-PE risk curve, a concentration of <50 nmol/L has been suggested [36]. However, specific threshold values at which the risk of PIH is increased may vary based on certain physiological attributes, including BMI, race, and gestational age, all of which may interfere with vitamin D status. It is, therefore, plausible that a range, rather than a specific cut-off point, for 25(OH)D exists in which women are less likely to develop PIH.

To date, a lack of evidence from long-term, high-dose vitamin D supplementation trials undermines our confidence around potential adverse effects associated with the prolonged use of high-dose vitamin D supplements [71]. However, obtaining trial data to specifically assess the risk of adverse outcomes at high concentrations is ethically challenging and likely requires an individual participant level (IPD)-analysis approach of past intervention trials with safety outcomes. In light of the increased risk of hypertension among women in Project Viva [61,62], and the uncertainty surrounding the concentration at which risk of hypercalcaemia increases, caution should be urged with regards to dosing, especially in pregnancy.

Our analysis of the literature clearly identifies the need to conduct a large dose-response RCT, considering race, season and background diet, including calcium intake, to determine whether improved vitamin D status will help protect against PIH. Implementation of such a trial must consider the dose-response relationship of serum 25(OH)D to total vitamin D intake [18]. In the absence of pregnancy-specific values, regulatory agencies currently recommend a minimum vitamin D intake of 400 IU/day [11,72] sufficient to protect against maternal deficiency at a threshold of 25–30 nmol/L, but this will not protect against neonatal deficiency at the same threshold [73]. It is now likely that ethical review boards may no longer approve a true placebo group in pregnancy trials with vitamin D, meaning future intervention studies might be required to screen at baseline for deficiency [74] or supplement a ‘low’ intake group, resulting in a minimum 25(OH)D concentration range of ≥30 nmol/L. For outcomes such as PIH, and PE in particular, where narrow ranges of 25(OH)D concentrations could be pivotal to determining a reduction in incidence rates, the absence of a true placebo group may pose further experimental challenges [18].

## 5. Conclusions

This review critically evaluates the current evidence for an association between vitamin D status and/or intake and risk of hypertensive disorders in pregnancy. The inclusion of both observational and interventional studies helped to avoid potential oversight in the published research as the limited quantity of data produced by interventional studies alone may limit our understanding of the state of the art with regard to vitamin D and PIH. While most studies report serum 25(OH)D concentrations, we also reported studies of maternal vitamin D intakes to ensure a comprehensive analysis. Another strength of this review is that it reports outcomes relating to all terms of PIH and is not limited to PE. Systematic reviews to date have been unable to draw firm conclusions regarding the potential of vitamin D to protect against gestational hypertensive disorders. Evidence from RCTs are limited to PE prevention and the current evidence base is weak and subject to a high risk of bias. Data from trials using combined vitamin D and calcium supplementation support a protective effect against PE among the supplemented groups. Observational cohort studies show a positive association between vitamin D deficiency and increased risk of PE, but results are hampered by suboptimal clinical phenotyping, incomplete subject characterisation and large heterogeneity between studies. Estimates of vitamin D intakes in observational studies are usually absent and, in those that report exposure levels, the overwhelming effect of micronutrient supplementation on vitamin D intakes opens the possibility of confounding by other nutrients. In light of evidence for a mechanistic role for vitamin D in the metabolic adaptation of healthy pregnancy, it is biologically plausible that the dysregulated metabolite concentrations in unhealthy pregnancies (including PE) may, in part, result from a deficiency in vitamin D [15]. We, therefore, extend the view expressed by several investigators, who stress a need for an adequately-powered, well-conducted RCT to establish a causal effect between vitamin D deficiency and increased risk of PE. 

## Figures and Tables

**Table 1:** Observational studies of serum 25(OH)D and risk of hypertensive disorders in pregnancy.

Author	Year	Design	*n*	Gestational Age	Outcome	Significant Association
Abedi [55]	2014	Case-control	118	Delivery	Risk of PE was higher at 25(OH)D concentrations <25 nmol/L (OR 24.04, 95% CI: 2.1, 274.8)	Yes
Achkar [40]	2015	Nested case-control	2144	<20 weeks	Risk of PE was higher at 25(OH)D concentrations <30 nmol/L compared to >50 nmol/L (OR 2.23, 95% CI: 1.29, 3.83)	Yes
Al-Shaikh [63]	2016	Cross-sectional	1000	Delivery	PIH was not seen at 25(OH)D concentrations ≥75 nmol/L but frequency of PIH was not significant	No
Álvarez-Fernández [44]	2015	Retrospective cohort	257	9–12 & 20–41 weeks	Risk of late onset PE was higher at 25(OH)D concentrations <50 nmol/L (OR 4.6, 95% CI: 1.4, 15)	Yes
Anderson [52]	2015	Case-control	48	First trimester	25(OH)D concentrations did not differ between preeclamptic/hypertensive and normotensive women	No
Bärebring [35]	2016	Prospective cohort	2000	First & third trimester	An increase in 25(OH)D of ≥30 nmol/L was associated with lower odds of PE (OR 0.22, 95% CI: 0.084, 0.581) but not PIH alone	Yes
Bodnar [58]	2014	Case-cohort	3703	≤26 weeks	Risk of severe PE was lower at 25(OH)D concentrations >50 nmol/L (RR 0.65, 95% CI: 0.43, 0.98)	Yes
Bomba-Opon [43]	2014	Prospective cohort	289	First trimester	25(OH)D concentrations were not related to early biomarkers of PE	No
Burris [61]	2014	Prospective cohort	1591	16.4–36.9 weeks	Higher 25(OH)D concentrations were associated with a greater risk of hypertension (OR 1.32 for per 25 nmol/L increment, 95% CI: 1.01, 1.72)	Yes
Gidlöf [49]	2015	Nested case-control	159	12 weeks	25(OH)D concentrations <50 nmol/L was not associated with PE	No
Hossain [38]	2011	Prospective cohort	75	Delivery	Women with 25(OH)D concentrations in the lowest vs. highest tertile were more likely to develop hypertension (OR 3.38, 95% CI: 0.40, 28.37) and/or PE (OR 2.28, 95% CI: 0.35, 23.28); ≤50 nmol/L was identified as the risk threshold	Yes
Kiely [39]	2016	Prospective cohort	1768	15 weeks	Risk of PE and small for gestational age combined was lower at 25(OH)D concentrations ≥75 nmol/L (OR 0.64, 95% CI: 0.43, 0.96)	Yes
Lechtermann [51]	2014	Nested case-control	63	Delivery	Mean ± SD summertime 25(OH)D status was lower in women with than without PE (45 ± 43 vs. 123 ± 73 nmol/L)	Yes
Mohaghegh [56]	2015	Case-control	91	Delivery	Mean ± SD 25(OH)D status was lower in women with than without PE (38 ± 34 vs. 58 ± 38 nmol/L)	Yes
Pena [54]	2015	Cross-sectional	179	Delivery	Preeclamptic mothers had a higher rate of 25(OH)D <50 nmol/L than those without PE (50% vs. 23%)	Yes
Ringrose [64]	2011	Case-control	187	Delivery	Hypertensive women had lower mean ± SD 25(OH)D concentrations compared with controls (62 ± 26 vs. 70 ± 29 nmol/L) in the univariate analysis but not when controlled for BMI	No
Robinson [53]	2013	Case-control	80	Diagnosis (28+ weeks)	Median 25(OH)D concentrations were lower in women with EOSPE than in controls (42 vs. 83 nmol/L)	Yes
Schneuer [50]	2014	Nested case-control	5109	10–14 weeks	25(OH)D status was not a predictor of PE	No
Scholl [41]	2013	Prospective cohort	1141	<20 weeks	Women with secondary hyperparathyroidism had a >2-fold increased risk of PE when 25(OH)D concentrations were <50 nmol/L (95% CI: 1.23-, 6.41-fold)	Yes
Shand [37]	2010	Prospective cohort	221	10–20 weeks	25(OH)D status was not related to PE	No
Singla [59]	2015	Case-control	174	NS (mean 35–36 weeks)	Mean ± SD 25(OH)D status was lower in women with than without PE (24 ± 12 vs. 37 ± 17 nmol/L)	Yes
Wei [36]	2012	Prospective cohort	697	12–18 & 24–26 weeks	25(OH)D concentrations <50 nmol/L at 24–26 weeks were associated with increased risk of PE (OR 3.24, 95% CI: 1.37, 7.69)	Yes
Wei [42]	2013	Prospective cohort	697	12–18 & 24–26 weeks	PlGF levels were lower in women with 25(OH)D concentrations <50 nmol/L	Yes
Wetta [58]	2014	Nested case-control	300	15–21 weeks	25(OH)D status in early pregnancy was not related to PE at <37 weeks’ gestation	No
Woodham [45]	2011	Nested case-control	164	15–20 weeks	For each 10 nmol/L increase in 25(OH)D, risk of severe PE decreased by 38% (95% CI: 0.51, 0.76)	Yes
Xu [47]	2014	Nested case-control	200	≥24 weeks	Risk of PE quadrupled when 25(OH)D concentrations were <37.5 nmol/L (OR 4.2, 95% CI: 1.4, 12.8)	Yes
Ullah [57]	2013	Case-control	188	>20 weeks	Risk of eclampsia and PE was higher at 25(OH)D concentrations <75 nmol/L (OR 5.14, 95% CI: 1.98, 13.37 and OR 3.9, 95% CI: 1.18, 12.87, respectively)	Yes
Zabul [46]	2015	Nested case-control	74	Late gestation	25(OH)D status did not significantly differ between preeclamptic and non-preeclamptic women	No

BMI, body mass index; CI, confidence interval; EOSPE, early onset severe preeclampsia; NS, not specified; OR, odds ratio; PE, preeclampsia; PIH, pregnancy-induced hypertension; PlGF, placental growth factor; RR, risk ratio.

**Table 2:** Observational studies of dietary vitamin D intake and risk of hypertensive disorders in pregnancy.

Author	Year	Design	*n*	Gestational Age	Outcome	Significant Association
Abedi [55]	2014	Case-control	118	Delivery	Vitamin D supplement use did not differ between preeclamptic and non-preeclamptic women	No
Anderson [52]	2015	Case-control	48	First trimester	Dietary vitamin D intake did not differ between hypertensive and normotensive women	No
Haugen [60]	2009	Prospective cohort	23,423	15, 22 & 30 weeks	Women taking a vitamin D supplement (400–600 IU/day) had a reduced risk of PE compared to non-users (OR 0.73, 95% CI: 0.58, 0.92)	Yes
Kazemian [65]	2013	Case-control	263	21–35 weeks	Vitamin D intake was not associated with risk of gestational hypertension	No
Oken [62]	2007	Prospective cohort	1718	First trimester	Women with higher vitamin D intakes had an increased risk of gestational hypertension (OR 1.11 per 100 IU, 95% CI: 1.01, 1.21)	Yes
Ringrose [64]	2011	Case-control	187	Delivery	Vitamin D intake (diet + supplements) did not differ between hypertensive and normotensive women	No

OR, odds ratio; CI, confidence interval; PE, preeclampsia.

**Table 3:** Summary of recent systematic reviews examining the relationship of maternal vitamin D status and/or intake and risk of preeclampsia.

Author	Year	Design of Studies	Number of Studies	Sample Size	Meta-Analysis (Yes/No)	Outcome	Association (Yes/No)
De-Regil [28]	2016	Randomised controlled trials	2	219	Yes	Relative to placebo, a ‘trend’ in the risk reduction of PE was seen among gravidae consuming supplemental vitamin D ^1^ (8.9% vs. 15.5%; average risk ratio 0.52; 95% CI: 0.25, 1.05)	Yes
De-Regil [28]	2016	Randomised controlled trials	3	1114	Yes	Combined supplementation of vitamin D ^1^ plus calcium resulted in a reduced risk of PE (5% vs. 9%; average risk ratio 0.51; 95% CI: 0.32, 0.80)	Yes
Arain [66]	2015	Intervention & observational	7	26,924	No	Risk of PE may be increased at lower levels of 25(OH)D (range < 37.5–75 nmol/L), but the relationship between vitamin D and PE is conflicted by large heterogeneity between studies	Yes
Harvey [67]	2014	Intervention & observational	12	642	Yes	Meta-analysis of 4 observational studies found the risk of PE did not increase with decreased vitamin D status ^1^ (pooled OR 0.75, 95% CI: 0.48, 1.19)	No
Aghajafari [26]	2013	Observational	9	3191	Yes	PE was significantly associated with 25(OH)D concentrations <50 nmol/L (pooled OR 1.79, 95% CI: 1.25, 2.58)	Yes
Hyppönen [30]	2013	Randomised trials	4	5982	Yes	Women receiving supplemental vitamin D ^1^ had a reduced risk of PE compared to controls (pooled OR 0.66, 95% CI: 0.52, 0.83)	Yes
Hyppönen [30]	2013	Prospective observational	6	6864	Yes	Mothers with higher serum 25(OH)D status ^1^ had a reduced risk of PE (pooled OR 0.52, 95% CI: 0.30, 0.89)	Yes
Hyppönen [30]	2013	Prospective observational	2	77,165	Yes	Mothers receiving supplemental vitamin D ^1^ in early pregnancy had lower odds of developing PE (pooled OR 0.81, 95% CI: 0.75, 0.87)	Yes
Tabesh [27]	2013	Observational	15	3007	Yes	Eight studies (2485 women) were included in the meta-analysis, for which PE was significantly correlated with 25(OH)D concentrations <50 nmol/L but not <38 nmol/L	Yes
Wei [29]	2013	Observational	8	2273	Yes	Risk of PE was increased at 25(OH)D concentrations <50 nmol/L (OR 2.09, 95% CI: 1.50, 2.90)	Yes
Christesen [68]	2012	Observational	9	24,704	No	Risk of PE was inversely associated with a vitamin D intake of ≥400–600 IU/day and/or status ≥37.5–80 nmol/L in studies where the number PE cases exceeded 40 but no association was found in studies with <40 cases of PE	Yes
Thorne-Lyman & Fawzi [69]	2012	Intervention & observational	7	NS	Yes	Pooled analysis of 2 studies, (>25,000 women), found no difference PE risk when stratified by highest and lowest categories of total vitamin D intake ^1^ (OR 0.95, 95% CI: 0.86, 1.06)	No
Nassar [70]	2011	Nested case-control	2	435	No	Sufficient evidence was not available to firmly established an association between first trimester 25(OH)D status and PE risk	No

^1^ Vitamin D dose range or status as defined in each included study. 25(OH)D, 25-hydroxyvitamin D; CI, confidence interval; NS, not specified; PE, preeclampsia; OR, odds ratio.
